# Exceptionally high rates of positive selection on the *rbcL* gene in the genus *Ilex* (Aquifoliaceae)

**DOI:** 10.1186/s12862-019-1521-1

**Published:** 2019-10-21

**Authors:** Xin Yao, Yun-hong Tan, Jun-bo Yang, Yan Wang, Richard T. Corlett, Jean-François Manen

**Affiliations:** 1 Center for Integrative Conservation, Xishuangbanna Tropical Botanical Garden, Chinese Academy of Sciences, Menglun, Mengla, 666303 Yunnan China; 2 Center of Conservation Biology, Core Botanical Gardens, Chinese Academy of Sciences, Mengla, 666303 China; 3 Southeast Asia Biodiversity Research Institute, Chinese Academy of Sciences, Yezin, Nay Pyi Taw, Myanmar; 4 Germplasm Bank of Wild Species, Kunming Institute of Botany, Chinese Academy of Sciences, Kunming, 650201 China; 5 CAS Key Laboratory of Tropical Forest Ecology, Xishuangbanna Tropical Botanical Garden, Chinese Academy of Sciences, Menglun, Mengla, 666303 Yunnan China; 6 Laboratoire de Systématique Végétale et Biodiversité, University of Geneva (retired), Chemin de l’Impératrice 1, CH-1292 Chambésy, Switzerland

**Keywords:** Rubisco, Positive selection, PALM, Environmental adaptation, Molecular adaptation

## Abstract

**Background:**

The genus *Ilex* (Aquifoliaceae) has a near-cosmopolitan distribution in mesic habitats from tropical to temperate lowlands and in alpine forests. It has a high rate of hybridization and plastid capture, and comprises four geographically structured plastid groups. A previous study showed that the plastid *rbcL* gene, coding for the large subunit of Rubisco, has a particularly high rate of non-synonymous substitutions in *Ilex*, when compared with other plant lineages. This suggests a strong positive selection on *rbcL*, involved in yet unknown adaptations. We therefore investigated positive selection on *rbcL* in 240 *Ilex* sequences from across the global range.

**Results:**

The *rbcL* gene shows a much higher rate of positive selection in *Ilex* than in any other plant lineage studied so far (> 3000 species) by tests in both PAML and SLR. Most positively selected residues are on the surface of the folded large subunit, suggesting interaction with other subunits and associated chaperones, and coevolution between positively selected residues is prevalent, indicating compensatory mutations to recover molecular stability. Coevolution between positively selected sites to restore global stability is common.

**Conclusions:**

This study has confirmed the predicted high incidence of positively selected residues in *rbcL* in *Ilex*, and shown that this is higher than in any other plant lineage studied so far. The causes and consequences of this high incidence are unclear, but it is probably associated with the similarly high incidence of hybridization and introgression in *Ilex*, even between distantly related lineages, resulting in large cytonuclear discordance in the phylogenies.

## Background

The genus *Ilex* (hollies) constitutes the monogeneric family Aquifoliaceae in the campanulid subclade of the asterids [2]. *Ilex* species are deciduous or evergreen dioecious trees or shrubs of mesic temperate, subtropical, tropical, and tropical-montane climates. At least 644 species have been described. *Ilex* has a near-cosmopolitan distribution, with the highest diversities in East-Asia, and in Central and South America, moderate numbers of species in Southeast Asia and North America, and a few in Africa, Australia, and Europe, and on most remote volcanic islands in the Atlantic and Pacific, including the Canary Islands, Madeira, Azores, Caribbean, Polynesia, Hawaii, New Caledonia, and Fiji [36]. The lineage is old, dating from the end of Cretaceous [36, 38], with the oldest accepted fossil record of 69 Million Years Ago (MYA, [31]). However, there is a large gap between this date and the estimated Miocene age of the most recent common ancestor of all extant *Ilex*, based on plastid and nuclear markers [36]. Many pre-Miocene fossils have been attributed to *Ilex* ([34]; [33]), suggesting extensive lineage extinctions, so that the extant genus is now phylogenetically isolated.

Abundant production of small, fleshy fruits, consumed primarily by birds in most species, can account for its wide dispersal, but the exceptional adaptive capacity in this morphologically relatively uniform genus is surprising. There is evidence for a high frequency of hybridization and introgression in *Ilex*, leading to a reticulate pattern of evolution. Phylogenetic trees obtained from nuclear and plastid data are very different [36] and Shi et al. [49] suggest that many of the 150–200 narrow-range species recognized in China may be a result of hybridization, which may also be true elsewhere. Widespread hybridization is supported by anatomical and morphological studies [3], and documented by molecular phylogenies [36–38, 47, 49].

A feature of the genus *Ilex* is that the *rbcL* gene, encoding the large subunit of Rubisco and a widely-used plastid sequence in phylogeny, appears to have a particularly high rate of non-synonymous substitutions compared with other genera [39]. This high rate of non-synonymous substitution suggests a positive selection on the plastid-encoded Rubisco large subunit in *Ilex*. Rubisco catalyzes the first steps of photosynthetic fixation of carbon and of photorespiration in all photosynthetic organisms (see [4, 10, 44], for recent reviews). The most prevalent form of Rubisco (form I) is an hexadecamer of 8 catalytic large subunits (L, harboring the active site) encoded by the *rbcL* gene in the plastome, and 8 regulatory small subunits (S), encoded in the nucleosome by a small family of *rbcS* genes [6]. The assembly and enzymatic function of Rubisco are, in part, guaranteed by specific interactions of the L subunit with the S subunits, assisted by several nuclear-encoded specific chaperones [22, 23].

Several studies provide evidence that positive selection on particular amino-acid residues of the Rubisco large subunit is involved in plant adaptation to various environmental stresses. This was proposed for the endemic Hawaiian genus *Schiedea* adapting to dry or wet habitats [28], in the heterophyllous aquatic plant *Potamogeton* [26], in switches from C3 to C4 photosynthesis [7, 30], in movements from dry to wet habitats in the Balearic Islands [15], in adaptation of *Cardamine resedifolia* to high altitudes [25], and associated with leaf traits and climate characteristics in the genus *Quercus* [24]. Moreover, studies based on a large sample of *rbcL* sequences show that positive selection is widespread in most lineages of land plants [21, 29].

A consequence of *rbcL* positive selection is a frequently observed pattern of coevolution between positively selected sites. Indeed, the multiple roles of amino acids in enzymes (folding, stability, enzymatic activity) imply that the modification of one parameter can affect other properties. Stability and activity of the enzyme are likely to be negatively correlated, and compensatory mutations are then needed to restore global stability [50]. Thus, when positive selection occurs on a residue, a compensatory co-evolving mutation on another residue of the molecule is likely to be detected. This has been confirmed in land plants [24, 25, 46, 53]. Rubisco has evolved CO_2_/O_2_ specificity ratios according to the environmental situation, allowing a perpetual fine-tuning of its performance [50]. Although the plastid *rbcL* gene sequence is highly conserved, this enzyme has played a role in plant adaptation to changing environments across geological time and continues to do so across recent geographical space.

Another feature of adaptive mutations in *rbcL* is that during introgression, plastid-encoded maternal L subunits of one species become associated with nuclear-encoded paternal S subunits of another species, potentially creating compatibility problems [48]. Similar compatibility problems could also arise from the numerous nuclear-encoded chaperones intimately linked to Rubisco subunits ([52]; [18]). The impacts and repair of such incompatibilities on Rubisco function should be most significant in lineages experiencing high rates of hybridization or/and introgression, as it is the case in *Ilex* [36].

In this study, we looked for evidence of positive selection on *rbcL* in the genus *Ilex*. We first investigate *rbcL* sequences (truncated after codon position 436) of 116 species from across the global range (“Worldwide dataset”) and then untruncated *rbcL* sequences of 124 accessions collected in natural habitats in China (“Chinese dataset”).

## Results

### Evolution of the gene *rbcL* of *Ilex* includes all the features of positive selection

#### Positive selection on the rbcL gene of Ilex

**Fig. 1:**
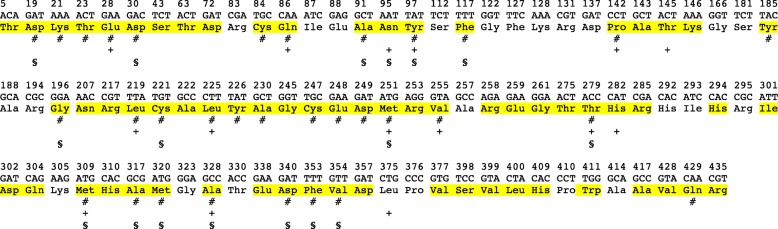
Variable codons (91 to 436) found among 116 *Ilex* sequences (Worldwide dataset) mapped on the accession *I. canariensis_90*, as reference. Uncolored codons are synonymous substitutions. Yellow codons are non-synonymous substitutions. **#** means positively selected codons (Bayesian posterior probability > 0.95 using the M8 model of Codeml, PAML (Table 1). **+** means positively selected residues found among the 20 most often positively selected residues of [29] (Table 1). **§** means residues involved in coevolution (Table 4)

The Worldwide dataset of 116 *rbcL* sequences (see Materials and Methods and Additional file 1: Table S1) included the first 436 codons. There were 91 variable codons, of which 24 had synonymous substitutions and 67 had non-synonymous substitutions (Fig. 1). In order to speed up the analyses, PAML calculations were conducted on *rbcL* sequences of the 4 *Ilex* plastid lineages [36] separately: the Asian/North-American Group 1 (14 sequences), the Asian/North-American Group 2 (36 sequences), the American Group 3 (31 sequences), and the Eurasian Group 4 (35 sequences). A 5th “20-Ilex” *rbcL* DNA matrix consisted of 20 selected species of *Ilex* representing the 4 plastid groups and a wide geographic distribution (see Fig. 3).

Positively selected sites were detected (at a Bayesian posterior probability of 0.95) by the program Codeml of PAML using model M8 [54]. Altogether for the five alignments studied, 32 positively selected residues were detected (Table 1). However, the SLR (Stepwise likelihood Ratio) test only confirmed 17 of these (Table 1). Using PAML, likelihood ratio tests (LRT) were done for each of the 4 alignments representing the 4 previously defined phylogenetic plastid lineages. Likelihood ratio tests of different models (model M2a allowing *ω* > 1 versus models M1a not allowing *ω* > 1, as well as model M8 allowing *ω* > 1 versus models M7 or M8a not allowing *ω* > 1) indicated that positive selection was highly probable for the 4 plastid groups at a significance level of < 0.001:

- Group 1, 2∆l = 42.88 (M2a/M1a), 49.68 (M8/M7), and 40.92 (M8/M8a).
- Group 2, 2∆l = 113.94 (M2a/M1a), 111.20 (M8/M7), and 111.04 (M8/M8a).
- Group 3, 2∆l = 52.14 (M2a/M1a), 52.16 (M8/M7), and 52.12 (M8/M8a).
- Group 4, 2∆l = 175.96 (M2a/M1a), 60.00 (M8/M7), and 42.74 (M8/M8a).

**Table 1:**
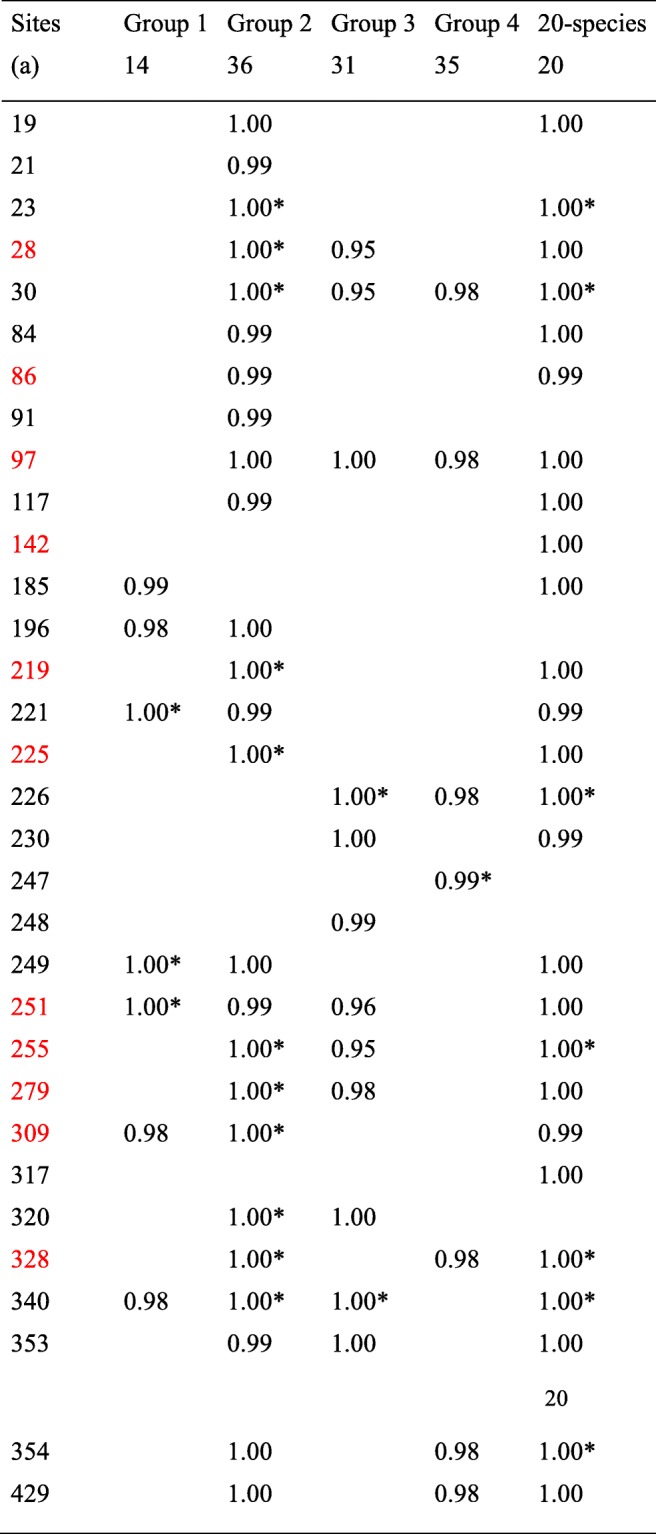
List of positively selected sites of *rbcL* of *Ilex* with posterior probabilities (PAML, Codeml, model M8) > 0.95 in the 5 subsets of the Worldwide dataset. Groups 1, 2, 3 and 4 represent the 4 phylogenetic plastid groups of [36] (Asian/North-American Group 1, Asian/North/American Group 2, American Group 3, and Eurasian Group 4. The 20-species sample represents a selection of the 116 specimens of [36], reflecting the 4 groups and a wide geographic distribution (see Fig. 3). In red, selected *Ilex* residues belonging to the list of the 20 most often positively selected sites detected in the 151 lineages of Kapralov and Filatov [29]

(*) indicates residues having ≥95% SLR support for positive selection

(a) Number of sequences

Positively selected sites were mainly found in the Asian/North American Group 2 (25 sites by PAML, 11 confirmed by SLR test), followed by the American Group 3 (12 sites, 2 confirmed), the Asian/North American Group 1 (7 sites, 3 confirmed), and then Eurasian Group 4 (7 sites, 1 confirmed) (Table 1).

A total of 25 positively selected residues were detected by PAML in the *rbcL* coding sequences of the Chinese dataset of 124 sequences (see materials and methods and Additional file 2: Table S2), six of which were new, of which residues 461 and 470 were unavailable in the Worldwide dataset truncated at residue 436 (Table 2). The SLR test confirmed 9 of these residues as positively selected. The Asian/North-American Group 2 again had the most positively selected residues (Table 2).

**Table 2:**
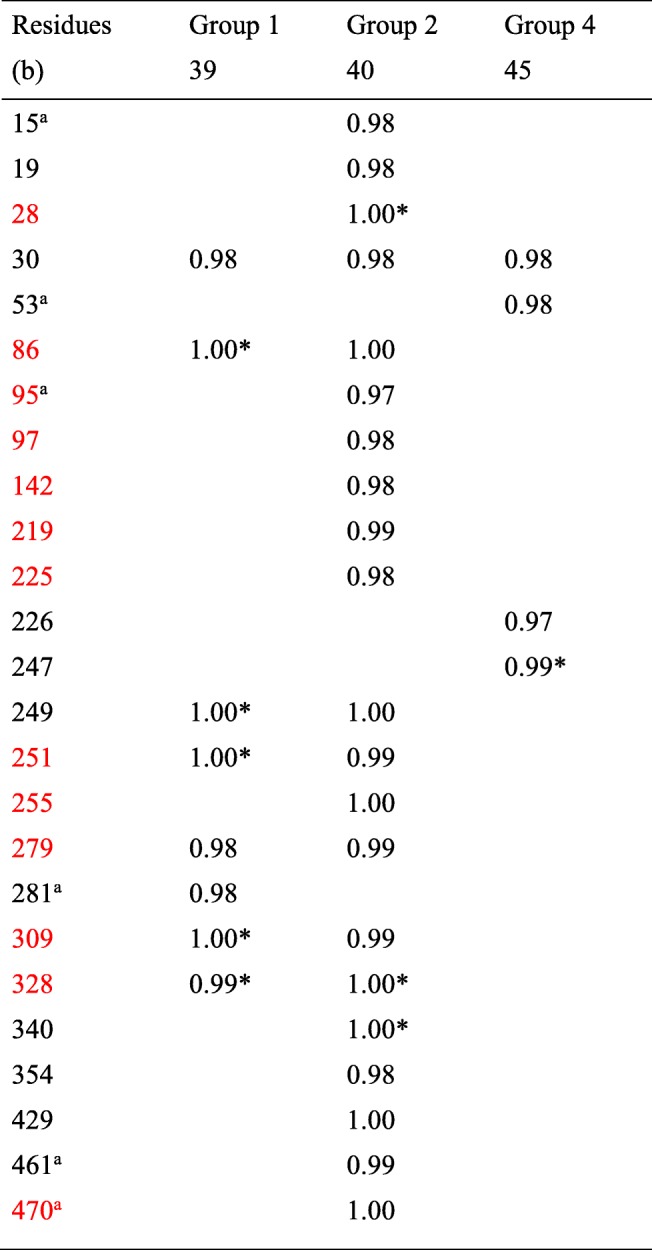
List of positively selected residues of *rbcL* with posterior probabilities (PAML, Codeml, Model M8) > 0.95 in the Chinese dataset. This dataset represents group 1, 2, and 4 (The American Group 3 is not present in China). In red, selected *Ilex* residues belonging to the list of 20 most often positively selected sites detected in the 151 lineages of Kapralov and Filatov [29]. (*) indicates that the residues have ≥95% SLR support for positive selection. (a) indicates that residues are not positively selected in the Worldwide dataset or that residues (461 and 470) were not available in the truncated Worldwide dataset. (b) Number of residues

#### Most positively selected sites are located at the surface of the folded rbcL

**Fig. 2:**
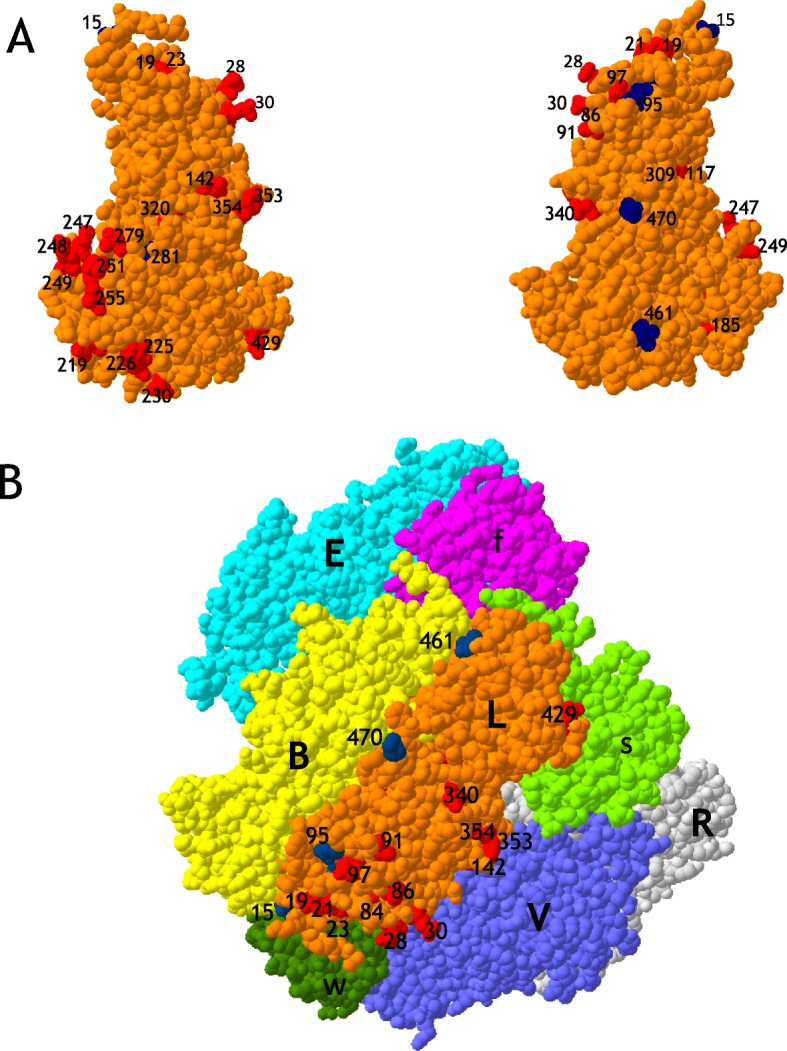
Positively selected residues of *Ilex* represented on the surface of the Rubisco large subunit. **a** Front and back of folded large subunit of the modeled *Ilex canariensis* 3-D structure. Visible positively selected residues from the Worldwide dataset are in red, while additional positively selected residues from the Chinese dataset are in blue. **b** Extrapolation on the Rubisco *Spinacia oleracea* 3-D structure (1RCX). Presented are the 4 large subunits (E,B,V and R) and 3 small subunits (f, s and w) that are in contact with one large subunit (here L, in orange). Positively selected residues are represented with the same colors as in A. Most of these residues (represented here on the L subunit) are exposed on the equatorial surface the L_8_S_8_ Rubisco hexadecamer. Sites 142, 429 and 461 are involved in intra-dimer, dimer-dimer and L/S interactions (see Table 3). All other sites (15, 19, 21, 23, 28, 30, 84, 86, 91, 95, 97, 340, 353, 354 and 470) represent positively selected residues potentially involved in interactions with chaperones

The 38 positively selected sites in *rbcL* detected by PAML (32 in the Worldwide dataset and 6 additional ones in the Chinese dataset) were mapped onto the 3-D structure of the modeled Rubisco large subunit of *Ilex canariensis* (see Materials and Methods). A large proportion of positively selected residues are located at the surface of the folded large subunit, with only 6 residues (53, 84, 196, 221, 317 and 328) being buried (Fig. 2a). Exposed residues could be candidates for interactions with other large subunits (intra-dimer and dimer-dimer interaction), with small subunits, and with Rubisco activase and other chaperones, as suggested by Kapralov and Filatov [29]. Extrapolating from the *Spinacia oleracea* Rubisco 3D-structure (1RCX, [51]), potential implications of *Ilex* positively selected residues in quaternary interactions were examined. Positively selected residues located within 6 Å (the van der Waals contact) of the surface of other subunits were selected using Swiss-Pdb Viewer (http://swissmodel.expasy.org). Some of these (13 of 38) were in van der Waals contact with another large subunit intra-dimer interface, a dimer-dimer interface, and/or with a small subunit (Table 3). Many positively selected residues of the equatorial surface of Rubisco were still visible (and potentially accessible by chaperones) even when the 4 interacting large subunits (B, E, R and V) and the 3 interacting small subunits (s, f and w) were represented in contact with the large L subunit (Fig. 2b). Positively selected residues 219, 225, 226, 429, and 461 of the large subunits were in contact with the small subunits (Table 3). The same was true for the numerous nuclear-encoded Rubisco chaperones that might interact with the many exposed positively selected residues on the equatorial surface of Rubisco (15, 19, 21, 23, 28, 30, 84, 86, 91, 95, 97, 340, 353, 354 and 470, see Fig. 2b), that were not in contact with any other L or S subunits (see for instance [22, 23]).

**Table 3:** Positively selected residues of the large subunit of *Ilex* Rubisco that are in contact with another L or S subunit, extrapolated from the *Spinacia oleracea* Rubisco 3D-structure (see Fig. 2). Thirteen positively selected residues of the Rubisco large subunit of *Ilex* are at the interface (≤ 6 Å) of intra-dimer (ID), of dimer-dimer (DD), and of large/small subunits (SSU). Two of them (219 and 461) share a contact with another large and with a small subunit. In brackets, the total number of residues of the Rubisco large subunit that are in contact with another large subunit or the small subunit in ID, DD and SSU interactions

Residues	Molecular interactions		
	ID (96)	DD (39)	SSU (76)
117	X		
142		X	
219		X	X
225			X
226			X
247	X		
248	X		
249	X		
251	X		
279	X		
309	X		
429			X
461	X		X

#### As expected, positively selected sites of the rbcL gene are extensively coevolving

Selecting only pairwise correlations above 0.5, CAPS detected 17 (out of 32) positively selected residues of the *rbcL* Worldwide dataset that were involved in coevolution (Table 4). Except for residue 95, all the others were detected as positively selected residues in the Worldwide dataset. However, residue 95 was detected as positively selected in the Chinese dataset (Table 2). The mean pairwise distance of coevolving pairs in the 3-D structure of the Rubisco large subunit was 22.9 Å.

**Table 4:** Pairs of coevolving residues detected by CAPS (Coevolution Analysis using Protein Sequences) in 116 *rbcL* sequences of the *Ilex* Worldwide dataset. Parameters used in CAPS are: alpha threshold 0.001, number of simulated alignments 1000, bootstrap value 0.95, await convergence and time correction on

Codon 1	Codon 2	Correlation	Bootstrap	Distance in Å
D 19	G 196	0.51	0.99	68.6
D 30	G 196	0.50	0.99	57.2
A 91	N 95	0.99	0.97	8.2
Y 97	M 309	0.70	1.00	12.8
F 117	T 279	0.67	0.98	13.2
C 221	M 251	0.51	1.00	20.9
M 309	A 328	0.70	1.00	19.7
A 317	M 320	0.62	0.98	6.7
A 328	D 340	0.78	1.00	16.9
A 328	V 354	0.70	1.00	23.9
D 340	V 354	0.70	1.00	21.0
F 353	V 354	0.73	1.00	5.5

### Compared with other lineages*, Ilex* has the highest rate of positive selection

Using the M8 model of PAML, positive selection on the *rbcL* gene of *Ilex* was compared with 8 lineages studied by Kapralov and Filatov [29] using recalculations based on their data. Eight of their lineages potentially have high rates of positive selection and were chosen for a comparison with *Ilex* (see their additional file 2). Three (out of 151) lineages (eurosids I-26, euasterids I-9 and Coniferales 6) had *d*N/*d*S values (in the “eleventh class” of the M8 model) higher than 20.5 (the value found in *Ilex*). Five (out of 151) other lineages (commelinids-7, commelinids-15, eurosids I-24, euasterids I-8 and Gnetales-1) had a higher proportion of detected positive selection sites (in the “eleventh class” of the M8 model) than 8.2% (the proportion found in *Ilex*).

PAML calculations indicated that the *rbcL* gene of the 20-species dataset of *Ilex* had 26 positively selected residues, while the largest number in the other 8 lineages was 15 in the commelinids-15 lineage (Table 5). All the others had < 10. The SLR test confirmed 7 positively selected residues in the *Ilex* 20-species dataset, while the highest number found in the other 8 lineages was 2 in the Coniferales-6 lineage. A comparison with other angiosperm lineages (Table 5), shows that the number of positively selected residues of *rbcL* in the Worldwide dataset of *Ilex* (32) (Bayesian posterior probability: 0.95) was never reached in any lineage studied by Kapralov and Filatov [29]. In their 151 studied datasets, the number of positively selected residues never passed 16 (see their additional file 3).

**Table 5:** Comparison of positive selection in *rbcL* of different lineages detected by PAML (M8 model) and by SLR (probability ≥0.95)

Lineages	N. seq^a^	dN/dS^b^	%p^c^	PALM^d^	SLR^d^
*Ilex*, the 20-species alignment	20	20.5	8.2	26	7
eurosids I-26 (Fagaceae)^e^	21	62.0 (61.0)	0.4 (0.4)	2 (2)	1
euasterids I-9 (Lamiaceae)^e^	19	27.9 (26.7)	6.3 (6.0)	3 (2)	0
Coniferales 6 (*Pinus*)^e^	35	13.1 (24.8)	1.6 (1.1)	6 (6)	2
commelinids-7 (Cyperaceae)^e^	27	1.1 (1.2)	12.7 (12.9)	0 (0)	0
Commelinids-15 (Poaceae)^e^	28	2.4 (2.4)	10.0 (9.9)	15 (15)	0
eurosids I-24 (Fagales)^e^	20	1.7 (1.6)	7.4 (8.5)	0 (0)	0
euasterids I-8 (Lamiaceae)^e^	20	2.7 (2.8)	10.7 (10.3)	2 (2)	0
Gnetales-1 (*Gnetum*)^e^	23	3.7 (3.5)	8.5 (8.6)	3 (3)	0
*Flaveria*	16	19.2	1.8	5	1
*Nothofagus*	24	6.2	5.4	7	1
*Populus*	14	5.1	3.9	6	0
*Potamogeton*	18	11.6	1.2	1	1
*Quercus*	30	18.4	0.6	2	1
*Salix*	28	2.5	9.7	1	0
*Schiedea*	27	26.1	0.6	0	0

^a^number of sequences

^b^mean dN/dS, “eleventh class” of the M8 model (PAML)

^c^proportion of positively selected residues, “eleventh class” of the M8 model (PAML)

^d^number of positively selected sites

^e^PAML re-calculations from data of Kapralov and Filatov [29], with their published results between brackets

The program Codeml of PAML was used in the above comparisons. It should be noted, however, that using the Site Likelihood-Ratio (SLR) test, a measure of the strength of evidence for selection, the number of positively selected residues remaining significant at 95% after the SLR adjustment was 17 (among the 32 residues found by PAML in the Worldwide dataset of *Ilex*, see Table 1). A similar decrease was generally observed in other studies when PAML results were adjusted by SLR (see for instance [27]). SLR adjustment did not change the major results of this study. The 17 positively selected residues in the Rubisco large subunit of *Ilex* detected by SLR still exceeded the maximum of 16 found in a broad survey of green plants by Kapralov and Filatov [29] using PAML.

Seven additional lineages (Table 5 and Additional file 3: Table S3) were also compared because they are trees (as are most *Ilex*) that have experienced frequent hybridization-introgression (*Nothofagus*, *Populus*, *Quercus*, *Salix*) or because of reported positive selection (*Flaveria*, *Potamogeton*, *Schiedea*). Positively selected sites are much more frequent in *Ilex* that in any other lineage studied (Table 5).

## Discussion

### A very high rate of positive selection on the *rbcL* gene of *Ilex*

This study has confirmed that the plastid gene *rbcL*, encoding the large subunit of Rubisco, has experienced an exceptionally high rate of positive selection in the genus *Ilex*. Kapralov and Filatov [29] listed the 20 most often positively selected *rbcL* residues, which accounted for > 70% of the cases of positive selection in their broad survey of green plants. Twelve positively selected residues detected in *Ilex* belonged to this top-20 list. Several residues detected in other lineages were also identified as positively selected in *Ilex* (Table 6). Thus, a large proportion of the *rbcL* sites positively selected in the green plants as a whole were also detected in the *Ilex*. This implies that the *rbcL* gene has a limited number of residue positions that can be positively selected for the fine-tuning of Rubisco [16], and that many of these have been selected in *Ilex*.

**Table 6:** Positively selected residues detected in *Ilex* (in both the Worldwide and Chinese datasets) also described in other lineages and associated with biological and environment traits

Residues	*Ilex* ^a^	Literature^b^	Biological and environmental traits	Authors
86^*^	Q to E,G	H,D to E,G	Rubisco kinetics	[16]
	Q to E,G	H to Y	Wet to dry environment	[28]
95^*^	N to S	N,T to D,S	Leaf traits and climate characteristics	[24]
	N to S	N.A.	Rubisco kinetics	[16]
142^*^	P to T	P to I,T,V	Specificity O2/CO2	[16]
	P to T	P to T	C3 to C4 plants	[50]
	P to T	P to A,T	C4 to C4 plants	[50]
	P to T	N.A.	C3 to C4 plants	[7]
219^*^	L to V	N.A.	Leaf traits and climate characteristics	[24]
225^*^	L to I	I to L	*Potamogeton* submerged, floating and terrestrial leaves	[26]
230	A to T	A to T	Wet to dry environment	[28]
281^*^	A to S	S to A	*Potamogeton* submerged, floating and terrestrial leaves	[26]
	A to S	A to S	Rubisco kinetics	[16]
309^*^	M to I	M to I	C3 to C4 plants	[27]
	M to I	M to I	Specificity O2/CO2	[15]
	M to I	M to I	C3 to C4 plants	[50]
	M to I	M to I	C4 to C4 plants	[50]
	M to I	N.A.	C3 to C4 plants	[7]
320	M to L	M to L	Rubisco kinetics	[16]
328^*^	A to S,G	A to S	Rubisco kinetics	[16]
	A to S,G	A to S	C3 to C4 plants	[50]
	A to S,G	A to S	C4 to C4 plants	[50]
	A to S,G	N.A.	C3 to C4 plants	[7]
	A to S,G	N.A.	Leaf traits and climate characteristics	[24]

^*^Ilex positively selected residues belonging to the 20-most often selected residues of Kapralov and Filatov [29]

^a^Amino acid substitution in *Ilex*

^b^Amino acid substitution in other lineages recorded in the literature

This particularly high rate of positive selection may affect the molecular clock of *rbcL* in *Ilex*, compared with other lineages. A high proportion of non-synonymous substitutions (such as positive selection) is indeed linked to a high rate of nucleotide substitution at the 1st and 2nd codon position compared to the rate of nucleotide substitution at the 3rd position. This last feature explains why the dating of the crown age of the extant genus *Ilex* using a fossil-calibrated molecular clock was at least two times overestimated using 1st and 2nd codon positions than using the 3rd position of *rbcL* [36]. As positive selection seems to be widespread in *rbcL*, a codon effect on age calculation using *rbcL* may be frequent ([45]; [35]), and this is particularly true in the genus *Ilex*.

### Positive selection and environmental traits in *Ilex*

Positive selection on *rbcL* is frequently observed in terrestrial plants but not in aquatic plants, algae, or bacteria [29]. It has been proposed that the thermal and water regime of the aquatic habitat is more stable, while land plants must adapt to the variability of their habitat, requiring a tuning of the activity of Rubisco by positive selection. The CO_2_/O_2_ specificity factor of Rubisco τ (determining the relative rate of photosynthesis and photorespiration) is very sensitive to cellular water availability, CO_2_ concentration, and temperature [14]. A relation between *rbcL* positively selected substitution at particular sites and morphological, biological, or climate characteristics of different lineages has been suggested (Table 6).

*Ilex* has two independent hotspots of speciation, in South America and East Asia, each comprising around 200 *Ilex* species. Both hotspots show high rates of recent speciation associated with, respectively, the final uplift of the Andes (from 5 to 2 MYA [19];, [1] [34];) and the Hengduan Mountains (4 to 3 MYA, [17]). In both of these hotspots, the recent development of high mountains and deep valleys may have accelerated the diversification of *Ilex* species through local vicariance, dispersal, isolation, secondary contact, and ecological speciation events [32, 42]. Asian/North-American Group 2 had the highest rate of positive selection on *rbcL*, but the species of this lineage included in this study do not occupy a wider range of environments than those of other lineages. This suggests that there is no simple relationship between positive selection and adaptability. Indeed, although *Ilex* occupies a wide range of environments, it is absent from areas without year-round soil moisture or with exposure to prolonged winter cold, which may have reduced the pressure for tuning Rubisco activity.

**Fig. 3:**
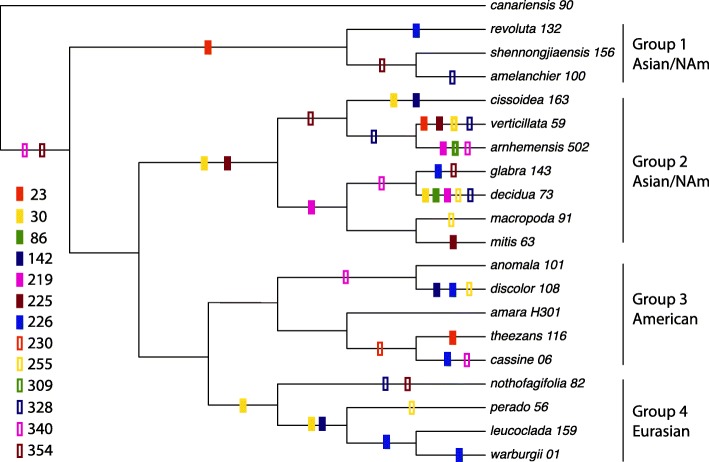
Mapping of positively selected sites in the 20-species tree. Only SLR positively selected sites are included, as well as *Ilex* positively selected sites recorded in Table 6. Asian/NAm means Asian/North American

The *rbcL* gene has a limited number of sites that can be positively selected. However, they are enough to suggest that cooperation of several different selected substitution sites can mediate a similar effect on Rubisco conformation. Conversely, one unique selected substitution can have different effects on *rbcL* folding and on Rubisco conformation, depending on the other sites of mutation [50]. Figures 3 shows that a particular selected mutation site of *rbcL* can be detected in different branches of the *Ilex* tree, indicating convergence in different clades and reversion in other, making interpretation rather complex. The fine-tuning of Rubisco is the result of a complex synergy of multiple coevolving substitutions sites. For instance, several positively selected residues (P142T, M309I and A328S) that were often associated to C3-C4 transition (Table 6) were also positively selected in *Ilex*, which is entirely C3. Most studies associating *rbcL* positive selection with a particularly biological or climatic trait involved only 2 or 3 selected mutations that were often linked by coevolution (for instance the pair M309I and A328S, [50]). The large number of positively selected *rbcL* sites found in *Ilex* suggests that other drivers are involved.

### Introgression may play a major role in the high rate of *rbcL* positive selection

Hybridization and introgression are pervasive in *Ilex* [36]*.* Advantageous positively selected residues in *rbcL* might contribute to the survival and spread of individuals and species with introgression. Adaptive positive selection on the Rubisco large subunit encoded by a non-recombining plastid DNA may promote, among other mechanisms (demographic events, fixation by chance), advantageous plastid captures [28]. This could be the case in *Ilex*. Huge cytonuclear discordances in the phylogeny of this genus have been reported: the plastid phylogeny is more geographically structured (with 4 geographically-determined phylogenetic clades) than the nuclear phylogeny, which was closer to the morphological classification of the genus [36]. Since the genus *Ilex* probably experienced extensive adaptation to changing habitats, plastid capture by adaptive introgression could well be promoted by a Darwinian selection on Rubisco.

On the other hand, such adaptive introgressions require that the surface of plastid-encoded Rubisco large subunits of one species fit with the surface of nuclear-encoded small subunits and chaperones of another species. This “molecular adaptation” could be essential for introgression between distantly related species, requiring a subsequent modification of molecular interactions. In *Ilex*, species from different lineages can hybridize when they come into secondary contact, helped by rather weak reproductive barriers and by many repeated intercontinental dispersals [36].

In this context, selection pressure on the Rubisco large subunit of one species might be, in part, the consequences such molecular incompatibility with the small subunit of another species. The Rubisco activase chaperone was suggested to interact with the region 89–94 of *rbcL* [43]. In *Ilex*, the exposed positively selected residues 91 and 95 may interfere with Rubisco activase (see Fig. 2b). Chaperones *rbcX* interacted with the C-terminal tail of one L subunit and the N-terminal region of another L subunit, to form 2 clamps that stabilized the dimer L_2_ [23]. Residues 461 and 470 (C-terminal tail) were positively selected. The same was true for positively selected residues 15 and 19 in the N-terminal region (see Fig. 2b).

It has to be noted that the majority of exposed positively selected residues of the *Ilex* Rubisco, found on the equatorial surface of the L_8_S_8_ hexadecamer shown in Fig. 2b, were not in the list of the 20 most often positively selected residues detected by Kapralov and Filatov [29]. These were residues 15, 19, 21, 23, 30, 84, 91, 340, 353 and 354 (see Fig. 2b and Tables 1, 2). Such uncommon positively selected sites, potentially interacting with chaperones, may represent a particularity of the genus *Ilex*. Thus, it can be postulated that the *rbcL* gene of taxa experiencing speciation by adaptive introgressions was expected to be under compensatory selective pressure to make the interface with nuclear-encoded partners compatible. In *Flaveria*, specific residue substitutions in the small subunit gene (rbcS) were correlated with the kinetic properties of Rubisco [30], but this observation was not further examined. In addition, Rubisco chaperones might have some effects on evolvability of *rbcL* [8].

Cytonuclear phylogenetic discordance is pervasive in *Ilex*, except in the well-defined Eurasian plastid group 4 which perfectly matches the nuclear clade Aquifolium [36]. Some hybridization events were noticeable between closely related members of this group. However, it appears that, for an unknown reason, introgression never occurred between species of the Eurasian group 4 and distantly related species belonging to the other groups 2, 3 and 4. This may explain the fewer positive selections *rbcL* in this group compared with other groups (Tables 1 and 2). Thus, in lineages experiencing introgression, a high rate of positive selection on the Rubisco large subunit may be expected. A large part of it would represent molecular adaptations resulting from introgression and plastid capture, a current situation in *Ilex*. Therefore, in future studies, molecular adaptations need to be discriminated from environmental adaptations of Rubisco (see [41]).

## Conclusions

In *Ilex,* the plastid *rbcL* gene coding for the large subunit of Rubisco had a higher rate of positive selection than in other lineages studied so far. Most of these positively selected residues are located at the surface of the large subunit and are likely to be involved in interactions with other plastid-encoded L or nuclear-encoded S subunits, as well as with the numerous nuclear-encoded Rubisco chaperones. Coevolution between positively selected sites to restore global stability is common. The high rate of positive selection in *Ilex* is probably linked with the pervasive hybridization and introgression in the genus, although the precise mechanisms behind this link are currently unclear.

## Materials and methods

We first investigated *rbcL* sequences of 116 specimens [36] representing most of the global distribution range of the genus (including 4 continents and 8 Atlantic and Pacific archipelagos); the “Worldwide dataset”. Then we examined *rbcL* sequences of 124 accessions of Chinese *Ilex* species recently collected in their natural habitats, mainly in Yunnan, SW China; the “Chinese dataset”. SW China is the center of *Ilex* diversity in East Asia and species occupy an exceptionally wide range of habitats, from lowland tropical rainforest to subtropical tree lines. Altogether, this study was based on 240 specimens.

### The worldwide dataset

For the detection of positively selected sites and site coevolution in *rbcL*, the data of Manen et al. [36], comprising 116 specimens and 108 species, was used. These 116 *rbcL* sequences were truncated after codon position 436. DNA-bank accession numbers are listed in Additional file 1: Table S1. To speed up PAML and SLR calculations (see below), the *rbcL* data was divided into five DNA alignments. Four of them represent previously recognized plastid groups ([36]; see Additional file 1: Table S1): Asian/North-American Group 1 (14 sequences), Asian/North-American Group 2 (36 sequences), American Group 3 (31 sequences), and Eurasian Group 4 (35 sequences). The fifth consisted of 20 representative *Ilex* species from the 4 phylogenetic plastid groups (species names in bold in Additional file 1: Table S1). All *Ilex* trees used in this study had the topology of the plastid tree of Manen et al. [36]. The *rbcL* sequence of the rather phylogenetically isolated *I. canariensis* [36] was chosen as a reference in alignments and trees because it didn’t belong to a particular plastid group. All alignments were done well using MUSCLE [9] because of the high conservatism in *rbcL* sequences. The five *Ilex* alignments (14 to 36 *rbcL* sequences) were in the size range of alignments used by Kapralov and Filatov [29] in their broad study based on 151 lineages of photosynthetic organisms, so their results could be compared with the results obtained in this study. For CAPS calculation (detection of coevolving residues, see below), the *rbcL* alignment of the 116 *Ilex* specimens was used.

### The Chinese dataset

New *rbcL* sequences from China consist of 124 accessions, including 77 identified and 35 as yet unidentified species. Most *rbcL* sequences were amplified and sequenced from DNA-extracts of fresh or dried material, using amplifying and sequencing primers of Fay et al. [13]. Some others were extracted from total plastid genomes of Yao et al. [55]. Additional file 2: Table S2 gives information on specimens and the DNA-bank accession numbers of their *rbcL* sequences. These sequences were not truncated after codon position 436. Several accessions that have not yet been morphologically determined were kept in the analysis because *rbcL* sequence comparisons with BLAST (Basic Local Alignment Search Tool, www.ncbi.nlm.nih.gov/BLAST*)* confirmed that they indeed represent *Ilex* and phylogenetic comparison with the sequences of Manen et al. [36] allowed the determination of the phylogenetic groups to which they belong: North-America/East-Asian Group 1 (39 sequences), Asian/North-American Group 2 (40 sequences), and Eurasian Group 4 (45 sequences). PAML and SLR calculations (detection of positive selection, see below) were done on these three plastid lineages separately and results compared with calculations on the Worldwide dataset.

### Detection of positive selection on *rbcL*

The nonsynonymous/synonymous rate ratio (*ω* = *d*_N_/*d*_S_) is an indicator of selective pressure at the protein level, with *ω* = 1 meaning neutral mutations, *ω* < 1 purifying selection, and *ω* > 1 diversifying positive selection. Amino acid sites in a protein are expected to be under different selective pressures and to have different underlying *ω* ratios. In the program Codeml of the PAML package (Phylogenetic Analysis by Maximum Likelihood [54];), different models that account for heterogeneous *ω* ratios among amino acid sites were tested by maximum likelihood on aligned protein-coding DNA sequences and the corresponding phylogenetic tree. The program is useful for testing adaptive molecular evolution and identifying amino acid sites under positive selection. The likelihoods obtained by the models M7 and M8a (allowing *ω* ≤ 1, the null hypothesis) and by the model M8 (allowing *ω* > 1) were compared by the likelihood ratio test (LRT). For the model M8, a Bayesian empirical procedure was used to estimate the mean value for each codon and the posterior probability that it is under positive selection. LRT of models Ma1 (the null hypothesis) and Ma2 (allowing *ω* > 1) were also calculated.

The use of PAML allows a comparison with earlier studies on *rbcL* positive selection, particularly Kapralov and Filatov [29]. The Sitewise Likelihood-Ratio test (SLR test, http://www.ebi.ac.uk/goldman-srv/SLR/#download), a measure of the strength of the evidence for selection ([40]), was used for adjustment. It distinguishes potential false-positive results that have been reported with PAML. Data on eight lineages from Kapralov and Filatov [29] were re-calculated using both PAML and the SLR test for comparison.

### Structural analysis of the large subunit of Rubisco

The protein structure of the large subunit of Rubisco for *Ilex canariensis* was modeled by homology using the Swiss Model server (http://swissmodel.expasy.org [5];). This program looks for the closest sequence from which a 3-D structure is available in the Protein Data Base (PDB) and uses it as model. The “magic fit” option of Swiss-Pdb Viewer shows that the three-dimensional structure of the large subunit of *Ilex canariensis* fits well with the three-dimensional structure of the large subunit of *Spinacia oleracea* (Additional file 4: Figure S1). Swiss-Pdb Viewer (http://www.expasy.org/spdbv; [20]) was used to localize positively selected residues on this modeled *Ilex canariensis* structure. Using the “Select” commands of Swiss-Pdb Viewer, the vicinity of positively selected residues at a 6 Å distance (the van der Waals contact) can be investigated at the interface between other Rubisco large subunits and between the large subunit and small subunits. As *rbcS* is not yet available in *Ilex*, an extrapolation was based on the *Spinacia oleracea* Rubisco three-dimensional structure (PDB accessions 1RCX, [51]).

### Detecting coevolving sites

CAPS (Coevolution Analysis using Protein Sequences, http://caps.tcd.ie [11, 12];) was used for identifying coevolution between amino acid sites. Blosum-corrected amino acid distances were used to identify amino acid covariation. Phylogenetic relationships were used to remove the phylogenetic and stochastic dependencies between sites. The 3D protein structure was used to identify the nature of the dependencies between coevolving amino acid sites [11]. Times of divergence were estimated as the mean number of substitutions per synonymous site. DNA alignment of *rbcL* sequences of the worldwide dataset of 116 *Ilex* specimens, the corresponding plastid ML-optimized tree of Manen et al. [36], and the previously determined 3-D structure of the large subunit of *Ilex canariensis* Rubisco, were used as inputs. Parameters were: alpha threshold 0.001, number of simulated alignments 1000, bootstrap value 0.95, await convergence and time correction on.

## Supplementary information

**Additional file 1: Table S1.** List of world-wide *Ilex* species [32]. In bold, species used in the 20-species analyses.

**Additional file 2: Table S2.** List of Chinese *Ilex* species studied here.

**Additional file 3: Table S3.** List of taxa used in comparison with *Ilex.*

**Additional file 4: Figure S1.** Comparison of the 3-dimensional structure of the Rubisco large subunit of *Spinacia oleracea* (in pink) with modeled 3-dimensional structure of the Rubisco large subunit of *Ilex canariensis* (in orange) using the “magic fit” option of Swiss-Pdb Viewer.

## Data Availability

All data included in the manuscript has been deposited in GenBank and their accession numbers can be found in supplementary files.

## Abbreviations

CAPS: Coevolution Analysis using Protein Sequences
GBIF: Global Biodiversity Information Facility
MYA: Million years ago
PAML: Phylogenetic analysis by maximum likelihood
PCA: Principal component analysis
PDB: Protein Data Base
Rubisco: Ribulose-1,5-bisphosphate carboxylase-oxygenase
SLR: Sitewise Likelihood-Ratio tests
