# Supplementary material for: Exceptionally high rates of positive selection on the rbcL gene in the genus Ilex (Aquifoliaceae)
Source: BMC Evol Biol. 2019 Oct 21;19:192. doi: 10.1186/s12862-019-1521-1 (PMC6805373; doi:10.1186/s12862-019-1521-1)

Supplementary Figure 1: Comparison of the 3-dimensional structure of the Rubisco large subunit of *Spinacia oleracea* (in pink) with modeled 3-dimensional structure of the Rubisco large subunit of *Ilex canariensis* (in orange) using the “magic fit” option of Swiss-Pdb Viewer.

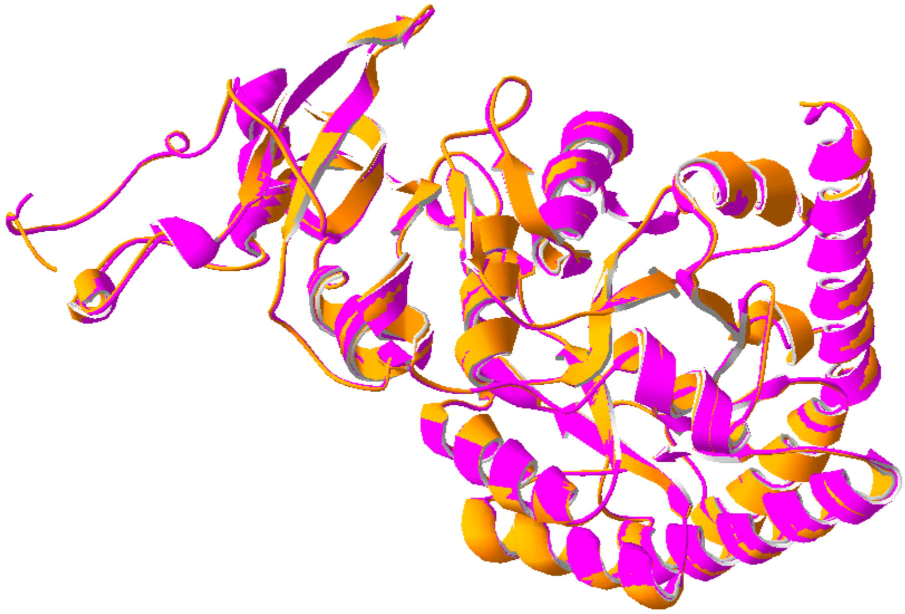

Supplement: Supplementary file 4 — Additional file 4: Figure S1. Comparison of the 3-dimensional structure of the Rubisco large subunit of Spinacia oleracea (in pink) with modeled 3-dimensional structure of the Rubisco large subunit of Ilex canariensis (in orange) using the “magic fit” option of Swiss-Pdb Viewer. [file 12862_2019_1521_MOESM4_ESM.pdf]
